# The FrogID dataset: expert-validated occurrence records of Australia’s frogs collected by citizen scientists

**DOI:** 10.3897/zookeys.912.38253

**Published:** 2020-02-17

**Authors:** Jodi J. L. Rowley, Corey T. Callaghan

**Affiliations:** 1 Australian Museum Research Institute, Australian Museum, 1 William Street, Sydney, New South Wales 2010, Australia Australian Museum Research Institute Sydney Australia; 2 Centre for Ecosystem Science, School of Biological, Earth and Environmental Sciences, University of New South Wales, Sydney, New South Wales 2052, Australia University of New South Wales Sydney Australia

**Keywords:** amphibians, bioacoustics, biodiversity data, citizen science, smartphone

## Abstract

This dataset represents expert-validated occurrence records of calling frogs across Australia collected via the national citizen science project FrogID (http://www.frogid.net.au). FrogID relies on participants recording calling frogs using smartphone technology, after which point the frogs are identified by expert validators, resulting in a database of georeferenced frog species records. This dataset represents one full year of the project (10 November 2017–9 November 2018), including 54,864 records of 172 species, 71% of the known frog species in Australia. This is the first instalment of the dataset, and we anticipate providing updated datasets on an annual basis.

## Introduction

### Citizen science biodiversity data

Biodiversity monitoring is critical for conservation, useful in warning of impending extinction crises, and has direct implications for management practices for improved biodiversity targets (Noss 1990; Pereira and Cooper 2006; Lindenmayer et al. 2012). The loss of funding, logistical constraints (e.g., time and spatial scale), and lack of interest by some government authorities in fully monitoring biodiversity make it important for other methods of biodiversity monitoring to be explored. For instance, citizen science (Silvertown 2009; Dickinson et al. 2012) is currently recognized as a method for achieving broad-scale biodiversity monitoring (Pocock et al. 2018; Callaghan et al. 2019). Citizen scientists are helping to assess various ecological and biodiversity aspects of birds (Sullivan et al. 2009), coral (Marshall et al. 2012), sharks (Vianna et al. 2014), and bees (Domroese and Johnson 2017), among other taxa. Additionally, some large-scale programs, such as iNaturalist (iNaturalist.org 2018) span various taxa.

### Frogs as sentinels of environmental change

Frogs and other amphibians are sensitive to changes in their environment due to their biphasic lifestyle (with most species having an aquatic larval stage and a terrestrial adult), semi-permeable skin, and reliance on specific environmental conditions for reproduction (Hopkins 2007; Lemckert and Penman 2012). Almost one-third of the 7,000 frog species known are at risk of extinction (Stuart et al. 2014; IUCN 2019), largely due to anthropogenic threats such as habitat loss and modification, disease, and invasive species. The implications are far-reaching, with frog populations declines shown to have large-scale, long-term ecosystem-level effects (e.g., Whiles et al. 2013).

Despite the need for biodiversity data on frogs, frogs are inherently difficult to survey, leaving a lack of detailed knowledge of broad-scale distributions, occurrences, and habitat associations. This is largely a result of logistical constraints, including a lack of funding available for surveys and access to often remote sites, and the fact that many frog species are difficult to detect, having activity patterns highly reliant on weather. Many frog species are also small and camouflaged, rendering them difficult to visually locate.

### Frog acoustic data

The frog advertisement call serves as a premating isolation mechanism (Blair 1964; Littlejohn 1969) and is therefore typically highly species-specific. As a result, advertisement calls are often used for frog species identification during surveys (Heyer et al. 2014) and in delineating species, including the description of new species (Littlejohn 1969; Rowley et al. 2016; Köhler et al. 2017). The identification of frog species via their advertisement calls may also minimise disturbances to the frog and its habitat.

All known frog species in Australia have audible advertisement calls and only a few are difficult to identify to species via their calls alone (e.g., several species of the genus *Pseudophryne* Fitzinger, 1843 in the places where they co-occur; Pengilley 1971). Further, several Australian frog species that are morphologically indistinguishable from related species can be identified to species by their calls (e.g., *Litoria jungguy* Donnellan & Mahony, 2004 and *Litoria myola* Hoskin, 2007). Although female frogs have been demonstrated to call in a handful of species (e.g., Goyes Vallejos et al. 2017), only male frogs are known to produce advertisement calls in Australia.

### Acoustic monitoring of frogs in Australia

Launched on 10 November 2017 and led by the Australian Museum, FrogID is the first citizen science initiative aimed at capturing validated biodiversity data on Australian frogs on a national scale (Rowley et al. 2019). The FrogID project collects data via a smartphone application allowing participants to submit recordings of calling frogs, which are then identified to species by experts (Rowley et al. 2019). If no frogs are heard calling (i.e., a FrogID user recorded an insect), submissions are identified as “Not a Frog”. If the recording is not sufficient to identify species (i.e., too short in duration, too much other noise in the recording), or there is an otherwise high level of uncertainty, the submission is identified as “Unidentified Frog”.

Publishing biodiversity data advances our collective knowledge on global biodiversity (Costello et al. 2013) and our ability to make informed conservation decisions. We hope that by making these occurrence data openly accessible (Michener 2015), others will find it useful, ultimately contributing to increased knowledge of Australia’s frogs and translating into increased conservation action. In this data paper, we detail the associated dataset.

## Project details

**Project title**: FrogID

**Sponsoring institution**: Australian Museum, 1 William Street, Sydney, NSW 2010

**Data published through GBIF**: https://doi.org/10.15468/wazqft

**Data published through a self-hosted Zenodo repository**: https://zenodo.org/record/3612700

### Funding

Funding for the FrogID project was provided by the Australian Government’s Citizen Science Grants program, the Impact Grants program of IBM Australia provided the resources to build the FrogID App. In-kind funding was provided by the Australian Museum. Bunnings and Fyna Foods are project partners.

### Data sensitivity

While effective conservation relies on accurate knowledge of where species occur, releasing the locations of observation records may have inadvertent negative impacts (Lindenmayer and Scheele 2017). Open locality information has resulted in the poaching of wildlife (Stuart et al. 2006), and particularly in the age of social media, access to precise locality data for certain species may also drive enthusiasts or wildlife photographers to locate, photograph or even remove species, sometimes resulting in habitat disturbance (Lindenmayer and Scheele 2017; Pike et al. 2010; Tulloch et al. 2018). A consideration of the potential impacts of publishing exact locality information is likely to be particularly important for FrogID records for three reasons: (1) FrogID occurrence data are derived from recordings of male frogs calling at breeding habitats, and habitat disturbance at these vital locations may influence breeding success; (2) visually locating or photographing frogs may disturb both the frog and breeding habitat, particularly for species that call from concealed microhabitats such as burrows (e.g. *Pseudophryne* and *Philoria* species); and (3) one of the major threats to frog species is disease, and pathogens may be transferred between individual frogs and between sites by people, representing a real risk to many frog species. For threatened frog species, or frog species with highly restricted distributions, revealing exact FrogID localities may therefore have serious, unintended negative consequences. Revealing exact localities for such species on private land may also result in trespassing (Lindenmayer and Scheele 2017).

We therefore follow ethical data publication guidelines (e.g., Chapman and Grafton 2008) and consider certain records as sensitive, thereby reducing geolocation accuracy in our publicly available dataset. We implement three geoprivacy options (Table 1) that take into account the state and national (DEE 2019) threat listings of the species, whether the species is range-restricted (i.e., has a geographic range or extent of occurrence of <20,000 km^2^), and whether the record falls within the known geographic range of these species (Table 2; Suppl. material 1). Further, because we provide the user id, the call id, and the time of every submission, for any submission which included either an obscured or private species, all species recorded in that submission also received the higher geoprivacy options. This means, for example, that some records of common and ‘open’ species are obscured. A total of 1,504 records’ coordinates for 74 species were therefore rounded to 0.1 degrees in this dataset. The complete dataset including sensitive information will be made available under licence to specific organisations and can be requested from the FrogID project.

**Table 1.** Geoprivacy options, which dictate whether or not the exact latitude and longitude coordinates are provided in our published dataset.

Geoprivacy option	Action
Open	No buffering of coordinates.
Obscured	Decimal coordinates rounded to nearest 0.1 degree. Actual coordinates are available upon special request.
Private	Record is not included in our published dataset but is available upon special request.

**Table 2.** Associated frog species threat categories and associated geoprivacy options (Table 1).

Frog species threat category	Geoprivacy
Not listed	Species is generally open, but may be obscured or private (if range-restricted or no confirmed recent records of the species).
Vulnerable	Species is generally open but may be obscured (with individual records outside of known range private), or private (if range-restricted or no confirmed recent records of the species).
Endangered	Species is generally obscured (with individual records outside of known range private) but may be private (if range-restricted or no confirmed recent records of the species).
Critically Endangered	Private.
Extinct	Private.

## Taxonomic coverage

**Figure 1.**
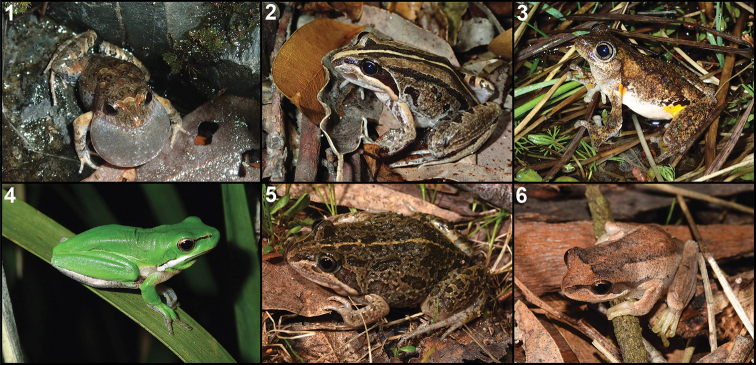
Photographs of the top six species recorded in the first year FrogID. **1***Crinia signifera***2***Limnodynastes peronii***3***Litoria peronii***4***Litoria fallax***5***Limnodynastes tasmaniensis***6***Litoria ewingii*.

**Figure 2.**
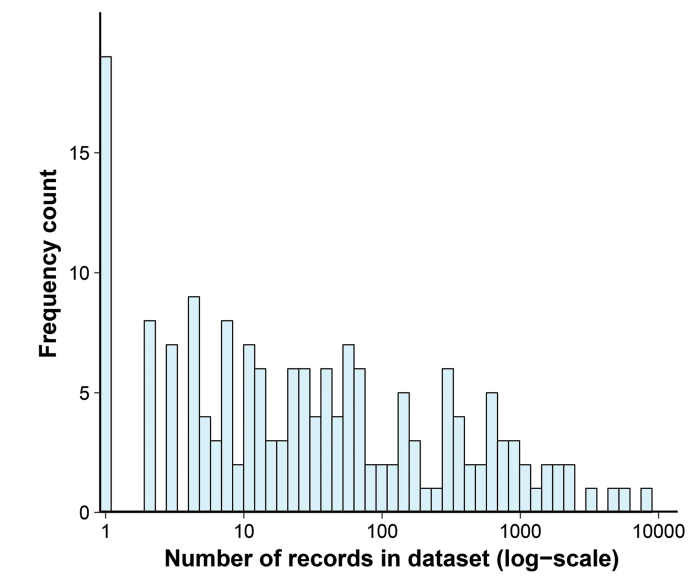
Frequency histogram for the 172 species published in our openly accessible dataset, showing the number of records (on a log-scale) and how many species have that associated number of records.

Throughout the first year of the FrogID project, 179 species of six families and 23 genera were recorded and are represented in the database, accumulating to 55,003 biodiversity records. The top-six most recorded species were: *Crinia signifera* Girard, 1853, *Limnodynastes peronii* (Duméril & Bibron, 1841), *Litoria peronii* (Tschudi, 1838), *Litoria fallax* (Peters, 1880), *Limnodynastes tasmaniensis* Günther, 1858, and *Litoria ewingii* (Duméril & Bibron, 1841) (Fig. 1). The number of records per species varied considerably, with the six most commonly recorded species accounting for almost half of all records (Fig. 2).

The openly accessible published dataset – after applying our aforementioned rules on sensitive species and records – hosts 172 species of the 179. A total of 139 submissions of 11 species were deemed private (Table 1), and as such, these records are removed from the published dataset. The seven species recorded by the FrogID project in the first year, but not published here are as all records were allocated a private geoprivacy status are: *Cophixalus aenigma* Hoskin, 2004, *Cophixalus concinnus* Tyler, 1979, *Cophixalus hosmeri* Zweifel, 1985, *Cophixalus monticola* Richards, Dennis, Trenerry & Werren, 1994, *Geocrinia alba* Wardell-Johnson & Roberts, 1989, *Geocrinia vitellina* Wardell-Johnson & Roberts, 1989, and *Litoria myola* Hoskin, 2007. The published openly accessible dataset consists of 54,864 records.

The frog fauna of Australia remains incompletely known. The database will be updated on an ongoing process, incorporating taxonomic changes, including any new species described. Annual releases will reflect these changes. The date of each data release will be critical for users to track.

### Taxonomic ranks

**Kingdom**: Animal

**Phylum**: Chordata

**Class**: Amphibia

**Order**: Anura

**Families**: Bufonidae, Limnodynastidae, Microhylidae, Myobatrachidae, Pelodryadidae, Ranidae

**Genera**: *Adelotus*, *Assa*, *Austrochaperina*, *Cophixalus*, *Crinia*, *Cyclorana*, *Geocrinia*, *Heleioporus*, *Lechriodus*, *Limnodynastes*, *Litoria*, *Metacrinia*, *Mixophyes*, *Myobatrachus*, *Neobatrachus*, *Notaden*, *Papurana*, *Paracrinia*, *Philoria*, *Platyplectrum*, *Pseudophryne*, *Rhinella*, *Uperoleia*

**Species**: *Adelotus brevis*, *Assa darlingtoni*, *Austrochaperina adelphe*, *Austrochaperina fryi*, *Austrochaperina gracilipes*, *Austrochaperina pluvialis*, *Austrochaperina robusta*, *Cophixalus australis*, *Cophixalus bombiens*, *Cophixalus infacetus*, *Cophixalus ornatus*, *Cophixalus saxatilis*, *Crinia bilingua*, *Crinia deserticola*, *Crinia flindersensis*, *Crinia georgiana*, *Crinia glauerti*, *Crinia insignifera*, *Crinia parinsignifera*, *Crinia pseudinsignifera*, *Crinia remota*, *Crinia signifera*, *Crinia sloanei*, *Crinia subinsignifera*, *Crinia tasmaniensis*, *Crinia tinnula*, *Cyclorana alboguttata*, *Cyclorana australis*, *Cyclorana brevipes*, *Cyclorana cultripes*, *Cyclorana longipes*, *Cyclorana maculosa*, *Cyclorana maini*, *Cyclorana novaehollandiae*, *Cyclorana occidentalis*, *Cyclorana platycephala*, *Cyclorana verrucosa*, *Geocrinia laevis*, *Geocrinia leai*, *Geocrinia rosea*, *Geocrinia victoriana*, *Heleioporus albopunctatus*, *Heleioporus australiacus*, *Heleioporus barycragus*, *Heleioporus eyrei*, *Heleioporus inornatus*, *Heleioporus psammophilus*, *Lechriodus fletcheri*, *Limnodynastes convexiusculus*, *Limnodynastes depressus*, *Limnodynastes dorsalis*, *Limnodynastes dumerilii*, *Limnodynastes fletcheri*, *Limnodynastes interioris*, *Limnodynastes peronii*, *Limnodynastes salmini*, *Limnodynastes tasmaniensis*, *Limnodynastes terraereginae*, *Litoria adelaidensis*, *Litoria aurea*, *Litoria barringtonensis*, *Litoria bicolor*, *Litoria brevipalmata*, *Litoria burrowsae*, *Litoria caerulea*, *Litoria chloris*, *Litoria citropa*, *Litoria cooloolensis*, *Litoria coplandi*, *Litoria cyclorhyncha*, *Litoria daviesae*, *Litoria dayi*, *Litoria dentata*, *Litoria electrica*, *Litoria eucnemis*, *Litoria ewingii*, *Litoria fallax*, *Litoria freycineti*, *Litoria gilleni*, *Litoria gracilenta*, *Litoria inermis*, *Litoria infrafrenata*, *Litoria jervisiensis*, *Litoria jungguy*, *Litoria latopalmata*, *Litoria lesueuri*, *Litoria littlejohni*, *Litoria meiriana*, *Litoria microbelos*, *Litoria moorei*, *Litoria nasuta*, *Litoria nigrofrenata*, *Litoria nudidigitus*, *Litoria olongburensis*, *Litoria pallida*, *Litoria paraewingi*, *Litoria pearsoniana*, *Litoria peronii*, *Litoria personata*, *Litoria phyllochroa*, *Litoria raniformis*, *Litoria revelata*, *Litoria rheocola*, *Litoria rothii*, *Litoria rubella*, *Litoria serrata*, *Litoria subglandulosa*, *Litoria tornieri*, *Litoria tyleri*, *Litoria verreauxii*, *Litoria watjulumensis*, *Litoria wilcoxii*, *Litoria xanthomera*, *Metacrinia nichollsi*, *Mixophyes balbus*, *Mixophyes carbinensis*, *Mixophyes coggeri*, *Mixophyes fasciolatus*, *Mixophyes fleayi*, *Mixophyes iteratus*, *Mixophyes schevilli*, *Myobatrachus gouldii*, *Neobatrachus aquilonius*, *Neobatrachus kunapalari*, *Neobatrachus pelobatoides*, *Neobatrachus pictus*, *Neobatrachus sudellae*, *Neobatrachus sutor*, *Neobatrachus wilsmorei*, *Notaden bennettii*, *Notaden melanoscaphus*, *Notaden nichollsi*, *Papurana daemeli*, *Paracrinia haswelli*, *Philoria kundagungan*, *Philoria loveridgei*, *Philoria pughi*, *Philoria richmondensis*, *Philoria sphagnicola*, *Platyplectrum ornatum*, *Platyplectrum spenceri*, *Pseudophryne australis*, *Pseudophryne bibronii*, *Pseudophryne coriacea*, *Pseudophryne dendyi*, *Pseudophryne douglasi*, *Pseudophryne guentheri*, *Pseudophryne major*, *Pseudophryne occidentalis*, *Pseudophryne raveni*, *Pseudophryne semimarmorata*, *Rhinella marina*, *Uperoleia altissima*, *Uperoleia arenicola*, *Uperoleia aspera*, *Uperoleia borealis*, *Uperoleia crassa*, *Uperoleia daviesae*, *Uperoleia fusca*, *Uperoleia inundata*, *Uperoleia laevigata*, *Uperoleia lithomoda*, *Uperoleia littlejohni*, *Uperoleia mahonyi*, *Uperoleia mimula*, *Uperoleia minima*, *Uperoleia mjobergii*, *Uperoleia rugosa*, *Uperoleia saxatilis*, *Uperoleia talpa*, *Uperoleia trachyderma*, *Uperoleia tyleri*.

## Methods

**Figure 3.**
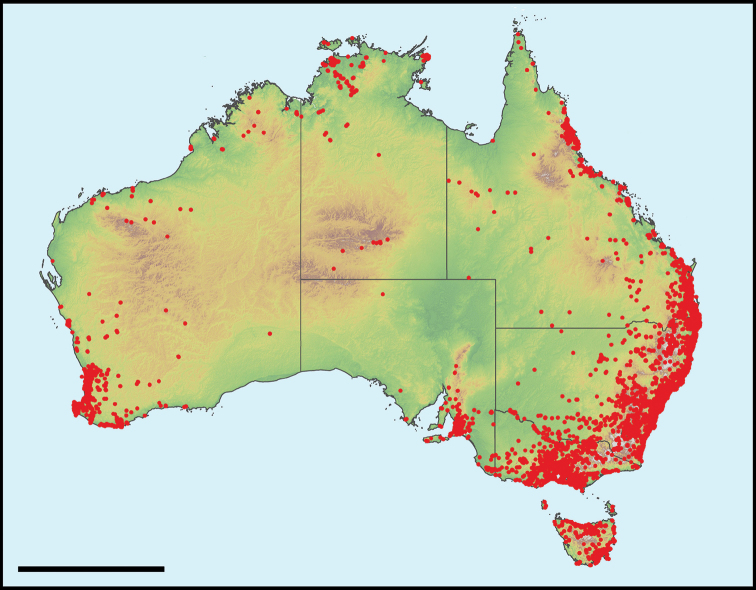
Occurrence records of calling frogs across Australia during year 1 of the FrogID project.

**Spatial coverage**: FrogID submissions have come from across Australia but, not surprisingly, are biased towards populated areas, with large areas of Australia, particularly in remote areas, lacking FrogID records. Despite this bias, the spatial coverage of this project encompasses the continent of Australia (Fig. 3), with frog records from 7,635,905 km^2^ (99%) of Australia’s landmass (measured as a minimum convex polygon enclosing all occurrences, excluding ocean).

**Temporal coverage**: FrogID is an ongoing data collection project, and this dataset (version 1.0) makes the first year of data collection available, 10 November 2017–9 November 2018. Data was exported from the FrogID database on 14 January 2020. We anticipate releasing an updated dataset annually.

**Validated frog records**: FrogID collects data via a free smartphone app (iOS and Android). Recordings are 20–60 seconds in MPEG AAC audio (mp4a) files. The time, date, and geographic location (latitude, longitude, and an estimate of precision of geographic location) are automatically added by the app at the time of recording. Each recording has an estimate of precision and, depending on the question, these records may influence results. As such, for records that rely heavily on geographic precision, we recommend filtering to records which have an estimate of geographic uncertainty of <3000 m. After recordings are submitted, they are stored in a cloud-based Content Management System (CMS), before being validated. FrogID validators, experts in identifying frog species by their calls, then use the audio and associated information, plus a reference call library, to identify the frog species calling in the recording. One submission can have multiple frog species calling within it. After these processes, we are left with a presence-only dataset of frog species in Australia. For a more detailed overview of methodology and design aspects, see Rowley et al. (2019).

## Dataset description

### Dataset specifications

**Object name**: FrogID dataset

**Character encoding**: UTF-8

**Format name**: Darwin Core Archive Format

**Format version**: 1.0

**Distribution**: https://doi.org/10.15468/wazqft; https://zenodo.org/record/3612700

**Publication date of data**: 22 January 2020

**Language**: English

**Licenses of use**: Creative Commons Attribution (CC-BY) 4.0 License

**Metadata language**: English

**Date of metadata creation**: 19 January 2020

**Hierarchy level**: Dataset

### Dataset description

The dataset includes basic biodiversity occurrence data, with Darwin Core terms (http://rs.tdwg.org/dwc/terms/), and is summarized in Table 3.

**Table 3.** Description of the data fields.

Data field	Description
datasetName	FrogID
basisOfRecord	Occurrence
dataGeneralizations	Highlights the geoprivacy options that were implemented
occurrenceID	Unique ID for each record in the dataset
sex	Male frogs are being recorded
lifestage	Adult frogs are recorded in FrogID
behavior	Only calling frogs are entered into the FrogID database
samplingProtocol	Call recording
country	Australia
machineObservation	An occurrence record based on an audio recording
eventID	Refers to the submission id – one submission can have more than one record
decimalLatitude	Latitude
decimalLongitude	Longitude
scientificName	Species name (*Genus species*).
eventDate	Date in year-month-day format
eventTime	Time the recording was taken
coordinateUncertaintyInMeters	A measure of the gps accuracy, measured in meters. See notes in methods
geoprivacy	Indicates whether the record is included and/or coordinates are buffered
recordedBy	Unique user id
stateProvince	Australian state of the record
modified	The date the record was last updated: useful for updating taxonomy or correcting errors in future dataset uploads

## Discussion

The FrogID database of expert-validated records of frogs across Australia represents a significant and growing contribution to our understanding of frogs in Australia. The first year of FrogID has resulted in the collection of over 55,000 expert-validated records of frogs across Australia. As frogs call almost exclusively from breeding sites, localities of calling frogs also provide vital information on their breeding habitats and times.

FrogID data provides a valuable resource aimed to help enhance our knowledge of frog distribution and occurrence in Australia. So far, the data have (1) shown new knowledge of distribution and breeding seasons for several species, (2) detected native frogs outside their native range, likely transported by humans, (3) collected data on invasive Cane Toads (*Rhinella marina*) in Australia, (4) and detected breeding populations of rare and threatened species (Rowley et al. 2019). We hope that by making these data available, researchers will capitalize on this unique dataset. There are growing statistical techniques to model presence-only data (Pearce and Boyce 2006), making it possible to assess species distribution models, phenology, diversity, and potentially abundance (Soroye et al. 2018) as statistical techniques relating to citizen science data continue to be developed.

## Supplementary materials

Supplementary material 1 The 241 frog species known from Australia (including the introduced Cane Toad), taxonomic authority and geoprivacy category used Data type: Species data https://binary.pensoft.net/file/380696This dataset is made available under the Open Database License (http://opendatacommons.org/licenses/odbl/1.0/). The Open Database License (ODbL) is a license agreement intended to allow users to freely share, modify, and use this Dataset while maintaining this same freedom for others, provided that the original source and author(s) are credited.Jodi J. L. Rowley, Corey T. Callaghan
